# Draft Genome Sequence of a Novel Bacterium, *Pseudomonas* sp. Strain MR 02, Capable of Pyomelanin Production, Isolated from the Mahananda River at Siliguri, West Bengal, India

**DOI:** 10.1128/genomeA.01443-17

**Published:** 2018-01-18

**Authors:** Vivek Kumar Ranjan, Tilak Saha, Shriparna Mukherjee, Ranadhir Chakraborty

**Affiliations:** a OMICS Laboratory, Department of Biotechnology, University of North Bengal, Siliguri, West Bengal, India; b Microbiology and Immunology Laboratory, Department of Zoology, University of North Bengal, Siliguri, West Bengal, India; c Department of Botany, Prasannadeb Women’s College, Jalpaiguri, West Bengal, India

## Abstract

The draft genome sequence of a novel strain, *Pseudomonas* sp. MR 02, a pyomelanin-producing bacterium isolated from the Mahananda River at Siliguri, West Bengal, India, is reported here. This strain has a genome size of 5.94 Mb, with an overall G+C content of 62.6%. The draft genome reports 5,799 genes (mean gene length, 923 bp), among which 5,503 are protein-coding genes, including the genes required for the catabolism of tyrosine or phenylalanine for the characteristic production of homogentisic acid (HGA). Excess HGA, on excretion, auto-oxidizes and polymerizes to form pyomelanin.

## GENOME ANNOUNCEMENT

The genome of *Pseudomonas* sp. strain MR 02 was sequenced using a NextSeq 500 system. Briefly, approximately 200 ng of DNA was fragmented by a Covaris M220 sonicator to generate ~400-bp segments. End-repaired products were size selected by AMPure XP beads, PCR amplified with index primers, and analyzed in a 4200 TapeStation system (Agilent Technologies). After obtaining the Qubit concentration, paired-end (PE) Illumina libraries, prepared using the Illumina TruSeq Nano DNA library preparation kit, were loaded onto the NextSeq 500 system for cluster generation and sequencing. The copied reverse strands were then used to sequence from the opposite end of the fragment. Thus, adapter-free data (1.1 Gb) were generated. The high-quality reads were then *de novo* assembled using the SPAdes genome assembler (1), and the quality of the assembly was evaluated using the QUAST software (2). The total size of the assembly (5,946,931 bp) was distributed in 90 scaffolds (average size of scaffolds, 66,077 bp; *N*_50_, 144,752 bp). Genes were predicted from the assembled scaffolds using Prokka version 1.12 (3). Functional annotation of the genes was performed using the NCBI Prokaryotic Genome Annotation Pipeline, yielding a total of 5,503 protein-coding genes, of which 5,460 have shown BLAST hits. Gene Ontology (GO) annotations of the genes were determined by the Blast2GO platform. GO assignments were used to classify the functions of the predicted genes. A phylogenetic tree was constructed with an MR 02 scaffold fasta file and its closely related species using the Alignment and Assembly Free (AAF) phylogeny tool. The phylogenetic tree was later uploaded to MEGA 6 (http://www.megasoftware.net/web_help_7/helpfile.htm#). The neighbor-joining (NJ) tree with a bootstrap of 500 showed that the most closely related strains of MR 02 were *Pseudomonas putida* DLL-E4 and *Pseudomonas monteilii* strain SB3101.

Pyomelanin originates from the catabolism of tyrosine or phenylalanine (4). A complete breakdown of tyrosine to acetoacetate and fumarate requires the enzymes aromatic amino acid transaminase (TyrB), 4-hydroxyphenyl pyruvic acid dioxygenase (HppD), homogentisate dioxygenase (HmgA), maleylacetoacetate isomerase (MaiA), and fumarylacetoacetate hydrolase (FahA). Two copies each of *tyrB* (aromatic amino acid transaminase) and *hppD* (4-hydroxyphenylpyruvate dioxygenase) genes, along with the genes of the *hmg* (homogentisate dioxygenase) operon (*maiA*, *fahA*, and *hmgA*), were present in the MR 02 genome. Interestingly, the MR 02 *hmg* gene sequence produced 98% (1,279/1,302 bp) identities with the *hmg* gene of *P. putida* strain DLL-E4 and *hmg* operon of *P. monteilii* strain SB3101, indicating several point mutations. In the absence of functional HmgA, or if homogentisic acid (HGA) production exceeds that of HmgA activity, HGA is overproduced and excreted from the cells (5, 6). Consequently, pyomelanin will be formed nonenzymatically outside the cell. The excreted homogentisic acid will form benzoquinone acetic acid on chemical oxidation and undergo self-assembly, yielding pyomelanin polymers.

### Accession number(s).

The GenBank accession number for the 16S rRNA gene sequence of MR 02 is MF401548. This whole-genome shotgun project has been deposited at DDBJ/ENA/GenBank under the accession number PESJ00000000. The version described in this paper is the first version, PESJ01000000. The sequence read data of assembled contigs have been deposited in the NCBI Sequence Read Archive under BioProject no. PRJNA415298. Strain MR 02 is currently available from the Korean Collection for Type Cultures (KCTC) under the accession number KCTC 62307.
